# A Novel Technique for the Topical Application of Botulinum Neurotoxin A to the Nasal Mucosa in Reducing Rhinitis Symptoms

**DOI:** 10.7759/cureus.75426

**Published:** 2024-12-09

**Authors:** Kathleen Chang, Jovian Wan, Song-Eun Yoon, Sky Wong, Kyuho Yi

**Affiliations:** 1 Aesthetics, Harmony Aesthetic Clinic, Adelaide, AUS; 2 Research, Medical Research Inc., Wonju, KOR; 3 Aesthetics, Brandnew Aesthetic Surgery Clinic, Seoul, KOR; 4 Aesthetics, Leciel Medical Centre, Hong Kong, HKG; 5 Anatomy, Yonsei University, Seoul, KOR

**Keywords:** botulinum neurotoxin, idiopathic rhinitis, mucosal atomizer, mucosal spray, persistent allergic rhinitis

## Abstract

Introduction: To date, no investigations have been published regarding the concentration, dose, and technique for a mucosal spray application of botulinum toxin A (BTA) to alleviate hypersecretory symptoms of rhinitis in humans. It is a promising option for reducing common symptoms of seasonal allergic rhinitis (AR) and idiopathic non-AR. It is safer and less painful than intranasal injections, with high reported satisfaction in reducing clinical symptoms.

Materials and methods: Seventeen patients with idiopathic rhinitis who presented to a private clinic in Australia were given 25 units of BTA (Letybo®, Hugel, South Korea) at a concentration of 50 U/mL in normal saline in each nostril via a mucosal atomizer device. At 4 and 12 weeks, a survey of symptom relief, satisfaction with treatment, and willingness to get a repeat treatment was collected.

Results: Patients were otherwise healthy, and the mean age was 40.1 ± 19.4 years. The majority (15 of 17) of subjects at 4 and 12 weeks reported significant symptom relief and high satisfaction, and were willing to get a repeat treatment. No adverse effects were reported.

Conclusion: Topical application of BTA on the nasal mucosa can be considered an effective therapeutic option in patients with persistent AR and idiopathic rhinitis. In comparison with injection techniques, BTA mucosal spray is effective at reducing persistent symptoms while minimizing the risk of pain and inadvertent systemic injection, and is able to promote contact with a wider mucosal area. Furthermore, it is easy to perform and is more tolerable for the patient. The authors present recommended concentrations, doses, and techniques.

## Introduction

Rhinitis is a common condition with allergic and nonallergic etiologies, affecting up to 40% of the population globally [1]. Characterized by inflammation of the nasal mucosa, it poses a significant public health challenge, impacting individuals across all age groups and geographic regions. Symptoms such as nasal congestion, obstruction, rhinorrhea, pruritus, and sneezing can be persistent and recurrent, profoundly impairing quality of life. These manifestations often result in sleep disturbances, daytime fatigue, and diminished productivity. The condition imposes substantial economic burdens, encompassing direct healthcare costs and indirect costs from absenteeism and reduced workplace performance. Additionally, chronic rhinitis has been increasingly recognized as a potential contributor to psychological distress, including anxiety and depression.

The pathophysiology of rhinitis is complex, involving diverse inflammatory mechanisms and neural pathways triggered by various stimuli, such as allergens, irritants, and environmental factors. Allergic rhinitis (AR) is primarily mediated by an immunoglobulin E-driven cascade of inflammatory events in response to specific allergens, whereas non-AR may be attributed to neurogenic inflammation and autonomic dysfunction [2]. The mechanistic heterogeneity of rhinitis underscores the need for individualized treatment approaches.

Current pharmacological management includes systemic agents such as oral antihistamines and corticosteroids, which remain the cornerstone of therapy. These are often complemented by topical treatments, including nasal sprays, decongestants, and immunotherapy. While these interventions provide symptom relief for many, their efficacy varies, and adverse effects such as drowsiness, dry mouth, and local irritation can limit patient compliance. For severe or refractory cases, surgical options, such as parasympathectomy of the pterygopalatine ganglion, are available but are typically reserved as a last resort due to their invasive nature and associated risks [1,2].

Despite the array of available treatments, many individuals with mild yet persistent symptoms fail to achieve satisfactory relief through allergen avoidance and conventional therapies. This unmet need has driven growing interest in alternative and complementary interventions. Among these, botulinum toxin A (BTA) has emerged as a promising treatment modality for rhinitis. Preliminary studies suggest that BTA, by modulating acetylcholine release and thereby influencing glandular secretion and local inflammation, may offer a novel mechanism for symptom alleviation [3-5]. The rising interest in BTA reflects a broader trend toward exploring innovative therapeutic options for patients who remain symptomatic despite standard treatment protocols.

## Materials and methods

This case series includes 17 patients aged 18-60 years who presented to a private cosmetic clinic in Adelaide, Australia, between January 2023 and February 2024. The study received ethical approval from the Yonsei University Medical Center Institutional Review Board (approval no. 2-2017-0023, dated June 22, 2017).

Participants were recruited for the study if they inquired about rhinitis or hay fever symptom relief. All participants were otherwise healthy, with no history of oral anticholinergic, steroid, antihistamine, or sedative use within the past three months. None had undergone prior nasal surgery or experienced nasal trauma. Informed consent was obtained from all participants for the treatment and inclusion in the study. Data collected included demographics (age and sex), symptom duration and type (see the Appendix for the symptom improvement questionnaire), symptom severity, seasonal and occupational factors, environmental exposures, and prior treatments.

The treatment protocol began with a nasal wash using normal saline. Patients were seated with their heads tilted upward at a 30° angle. BTA was prepared by reconstituting 100 units of letibotulinumtoxin A (Letybo®, Hugel, South Korea) in 2 mL of normal saline. A total volume of 1 mL (50U) was drawn up into a 1 mL low dead-space syringe (Omnifix®-F, B. Braun, B. Braun Medical Inc., Melsungen, Germany). A mucosal atomizer device (MAD Nasal™, Teleflex Medical, Wayne, PA) was attached to the end of the syringe, and each nostril was sequentially sprayed with 0.5 mL of the solution. Thus, 25U of BTA was delivered to each nostril. Patients were instructed not to swallow any fluid that dripped into the throat and to maintain their position for 30 seconds. Postprocedure care included refraining from nose blowing, eating, or drinking for one hour. They were instructed to avoid any prescription medications for rhinitis for 12 weeks to assess the effect of the current intervention.

Data presentation and analysis

Data collected from the participants were analyzed using descriptive statistics to summarize patient demographics, symptom types, and treatment outcomes. Symptom improvement was measured by comparing baseline scores with follow-up evaluations at 4 and 12 weeks. The responses to the symptom improvement questionnaire were categorized into "improvement" and "no improvement." A simple percentage analysis was used to present the proportion of participants reporting symptom improvement. Additionally, the level of satisfaction and willingness to undergo repeat treatment were recorded as binary outcomes (yes or no). All statistical analyses were performed using appropriate software to ensure accuracy and validity.

Follow-up evaluations were conducted at 4 and 12 weeks via telephone interviews, face-to-face consultations, or email. Participants completed a questionnaire assessing symptom improvement from baseline (options: yes, no, or unsure), treatment satisfaction (yes or no), and willingness to undergo repeat treatment (yes or no).

## Results

The mean age of the participants was 40.1 ± 19.4 years, with a range of 19-60 years. Ten subjects were female, and seven were male. All individuals had previously trialed at least one prescription medication as directed by a physician. Ten patients (58.8%) attributed their clinical symptoms to seasonal allergies, while the remaining seven were unable to identify a clear cause. The reported symptoms included rhinorrhea (15/17), nasal congestion (16/17), sneezing (13/17), foreign body sensation (5/17), and pruritus (15/17). Four patients had a formal diagnosis of asthma. Fourteen participants had at least one previous exposure to botulinum toxin in the form of cosmetic injections throughout their lifetime.

Questionnaire responses were obtained from all participants at 4 and 12 weeks after treatment. At the four-week follow-up, 16 of 17 participants reported an improvement in symptoms, while one individual reported no change. By 12 weeks, 70% (12 participants) continued to report symptom improvement compared to baseline, whereas five participants indicated no improvement. All participants who experienced symptom improvement at four weeks (16/17) expressed satisfaction with the treatment and willingness to undergo repeat procedures (Table 1). No adverse events, such as epistaxis or dysphagia, were reported at either the 4- or 12-week follow-up.

**Table 1:** The self-survey of symptom improvement

Questions	Week 4	Week 12
Have symptoms improved from baseline?	Yes, 16 (94%) No, 1 (6%)	Yes, 12 (70.6%) No, 5 (29.4%)
Would you get the treatment again?	Yes, 16 (94%) No, 1 (6%)	Yes, 16 (94%) No, 1 (6%)

## Discussion

Rhinitis can be clinically classified into allergic and nonallergic types [1]. Patients with persistent or seasonal symptoms increasingly seek treatments beyond conventional over-the-counter and prescription medications. In certain cases, specialized investigations are undertaken to identify pathological hallmarks such as mucosal inflammation, eosinophilia, vasodilation, or glandular hyperplasia [5].

The pathophysiology of rhinitis symptoms is well documented in the literature. In human studies, injections of BTA into the nasal cavity have consistently shown efficacy in alleviating symptoms of both allergic and non-AR [6-11]. Research exploring injection sites, including the middle and inferior turbinates and submucoperichondrium of the septum, revealed symptom improvement comparable to that achieved with antihistamines and intranasal steroids. A wide range of doses was investigated in the injection studies, ranging from 2 to 30 units per nostril [12-16]. However, evidence supporting topical application remains limited [15,17,18]. Zand et al. [19] investigated a gelfoam impregnated with BTA placed intranasally for one hour, demonstrating improvements in nasal secretions, patency, congestion, loss of smell, and pruritus.

Despite the growing popularity of BTA for rhinitis treatment, often referred to as "Haytox" in regions such as Australia and the UK [4], no published studies have explored the use of a mucosal spray application. This method offers a simpler and less invasive alternative to injections, potentially achieving comparable efficacy while minimizing the risk of systemic absorption. Due to BTA being a more expensive option compared to conventional therapy, it may be reserved for patients who wish to improve their quality of life and consent to the cost of treatment.

This study aims to establish an optimal dose and concentration of BTA for in-office mucosal spray application. By doing so, it seeks to provide clinicians with a safe, effective, and patient-friendly option for managing persistent symptoms of rhinitis.

This preliminary study shows that when 25U of BTA is administered to each nostril, the majority (96%) of participants reported symptom improvement at four weeks. However, it has several limitations that should be considered. First, the sample size was relatively small (17 patients), which may limit the generalizability of the findings. Additionally, the study lacked a control group, making it difficult to definitively attribute the observed improvements to the treatment rather than to natural variation or other confounding factors. The study also relied on subjective self-reported data, which may be influenced by patient bias or recall error. Finally, the follow-up period was limited to 12 weeks, and longer term outcomes remain unclear. Future studies with larger sample sizes, randomized controlled designs, and extended follow-up periods are necessary to further validate the efficacy and safety of mucosal spray BTA for rhinitis treatment. Future research will elucidate optimal dosing for efficacy, variables such as sex or symptom severity that affect outcome, and objective measures such as histopathological changes before and after the intervention.

## Conclusions

This preliminary study investigates the use of mucosal spray BTA for alleviating symptoms of both allergic and non-AR. The results suggest that mucosal spray BTA is an effective and well-tolerated treatment option for patients with persistent rhinitis symptoms, providing symptom relief for up to 12 weeks. By offering a less invasive, patient-friendly alternative to traditional injection methods, mucosal spray BTA may provide significant advantages in terms of convenience and patient compliance. High levels of satisfaction and a willingness to undergo repeat treatments underscore the potential of this approach as an alternative to conventional therapies. However, further studies with larger patient populations and longer follow-up periods are required to confirm the long-term efficacy and safety of this treatment. As the landscape of rhinitis management evolves, novel treatment modalities such as BTA could play a critical role in improving patient outcomes, particularly for those who are refractory to standard therapies.

## Appendices

Symptom improvement questionnaire

1. Did you experience an improvement in your rhinitis symptoms following treatment? Yes No Unsure

2. Please indicate which symptoms improved after treatment: Nasal congestion Rhinorrhea Sneezing Pruritus Foreign body sensation None of the above

3. Overall, how satisfied are you with the treatment? Very satisfied Satisfied Neutral Dissatisfied Very dissatisfied

4. Would you be willing to undergo repeat treatment? Yes No
